# Culture Method and PCR for the Detection of *Helicobacter pylori* in Drinking Water in Basrah Governorate Iraq

**DOI:** 10.1155/2012/245167

**Published:** 2012-06-21

**Authors:** A. A. Al-Sulami, T. A. A. Al-Edani, A. A. Al-Abdula

**Affiliations:** ^1^College of Education, University of Basrah, Ashar P.O. Box 2108, Ashar, Basrah, Iraq; ^2^Department of Biology, College of Sciences, University of Basrah, Ashar P.O. Box 2108, Ashar, Basrah, Iraq

## Abstract

*Helicobacter pylori* is recognized by the World Health Organization to be the primary cause of peptic ulcers, chronic gastritis, and stomach cancer, though the source of human infection is not well understood. One of the problems in understanding the source of human contamination is the difficulty in isolating the organism from the environment. However, the combination of PCR results with those of culturing of 471 drinking water samples can provide a more accurate picture of *H. pylori* detection. In this method 78 presumptive *H. pylori* colonies out of 266 tap water samples were obtained in the preliminary detection on modified Columbia agar (MCUA) slant relying on urease positivity with a rate of 29.3%. However, only 11 out of them were confirmed by Gram staining and biochemical tests reducing the rate to 4.13% whereas only 3 (1.46%) from 205 reverse osmosis (RO) water samples. Furthermore, only 6 (54.5%) out of the 11 isolates from tap water and 1 (33.3%) of the 3 RO isolates were confirmed by 16SrRNA PCR. Thus PCR confirmation reduced the rate to 2.2%. In addition, only 4 (4%) of 100 tap water samples negative for *H. pylori* by culture method were *H. pylori* positive by 16SrRNA. Water samples were collected from 24 districts of Basrah Governorate from February–December 2009. The direct recovery of *H. pylori* from drinking water is both alarming and scientifically exciting in terms of the investigation of its epidemiology.

## 1. Introduction

*Helicobacter pylori* is recognized as the major cause of gastritis and peptic ulcer and gastric mucosa-associated lymphoid tissue (MALT) gastric lymphoma [1]. The mechanism of *H. pylori* pathogenic effect is unclear but is believed to be related to host bacterial interactions initiated by virulence genes, and it is possible that these effects are enhanced by invasiveness of the bacterium [2–5]. *H. pylori* changes from the normal spiral-shaped bacillary form into the coccoid form when it is exposed to water or to other adverse conditions [6]. Hence attempts have been made to develop artificial media to achieve better culture recovery results than those obtained from traditional Columbia blood agar [7, 8]. Polymerase chain reaction (PCR) methods have also been used to detect *H. pylori* as its 16SrRNA gene sequence analysis unambiguously differentiated the *Helicobacter* genus from the closely related *Campylobacter* genus and other *Helicobacter *species [9]. The presence of *H. pylori* in drinking water which was detected by PCR has been reported from several countries [10–12]. Hegarty et al. [13] also demonstrated the presence of respiring *H. pylori* from US surface water. The prevalence of disease attributed to *H*. *pylori* in Iraq is not available despite of its commonality. 

Basrah Governorate, where Basrah city is located, has a population of about three millions; its water supply is mainly derived from three sources, Shatt-Al-Arab River, Tigris River, and Bada lake. Water from these sources is treated at 22 treatment works and distributed through approximately 13,000 Km pipe network.

Since the 1980s there has been a general marked deterioration in water quality in Iraq, reflecting the environmental degradation of the country caused by successive armed conflicts. 

The aim of this study was isolating *H. pylori* from drinking water in Basrah, Iraq, on modified Columbia urea agar (MCUA) and HP media using MDCS method [7] and then confirming that by conventional biochemical tests and 16SrRNA PCR.

## 2. Methods

### 2.1. Sample Collection and Culturing

266 samples of tap water and 205 samples from tankers supplying Reverse osmosis (RO) were collected from 24 districts covering more than 90% of Basrah Governorate during the period from February 2008 to December 2009. Samples of 500 mL water each were collected in sterile glass flasks and examined for chlorine concentration using *o*-toluidine. Samples were transferred within 1-2 hr. to the laboratory and filtered through 0.22 *μ*m Millipore filter membrane. Each membrane was then immersed into 2 mL of tryptic soy broth (TSB) for 1 h. After that each 2 mL TSB was taken and placed at the lower portion of the slanted MCUA tube. Each tube was tilted a few times to allow the added broth to spread bacteria on the upper part of the slant. Slanted MCUA tube, was incubated microaerophically at 37°C for 1-2 days, after which color changes from orange to pink in the solid phase, indicating urease activity. The resulting system is a simple monophasic-diphasic culture setup (MDCS), a diphasic solid liquid environment at the lower part of the test tube and a monophasic solid one above it [7]. From the bottom and the upper portions of the slanted MCUA tube subcultures were done on plates of MCUA and HP media for purification. 

No controls were used in the isolation of the strains and also in PCR as they are out of reach for us in Iraq. 

### 2.2. Primary Diagnosis of H. pylori

The suspected purified colonies were chosen according to the Gram staining and cultural characteristics.

### 2.3. Biochemical Tests

Biochemical tests include production of catalase, oxidase, urease, and H_2_S, nitrate reduction, growing in 3.5% NaCl, growing with 1% glycine, and growing at different temperatures.

### 2.4. Antibiotic Sensitivity Test

The method of Piddock [14] was used to test the sensitivity of 14 isolates of *H. pylori *from drinking water to seven types of antibiotics, kanamycin 30 *μ*g, erythromycin 15 *μ*g, tetracycline 30 mg, ampicillin 10 *μ*g, rifampicin 5 *μ*g, amoxicillin 30 *μ*g and gentamycin 30 *μ*g (Bioanalyse, Turkey).

### 2.5. 16SrRNA Identification of Isolates

All isolates from tap and RO water samples which gave positive results by biochemical tests as *H. pylori *and (100) samples which were *H. pylori* negative by culture method were further confirmed by using primers specifically designed for the identification of *H. pylori *based on 16SrRNA sequence [15]. The primers for 500 bp product of the 16SrRNA sequence are represented by the forward primer sequence: 5 GCT AAG AGA TCA GCC TAT GTC C3 and the reverse one: 5 TGG CAA TCA GCG TCA GGT AAT G3.

### 2.6. Preparation of Bacterial Genomic DNA

Genomic DNA from each isolate was prepared by vortex after suspending a loopful of colonies in 1 mL of phosphated-buffer saline (PBS) 7.6, centrifuging at 14000 ×g for 2 min, and boiling the pellet in 1 mL of distilled water for 1 min [16]. The samples were then centrifuged at 12000 ×g for 4 min at 4°C and the supernatants were stored in sterile vials at −70°C until they were used as PCR templates. Genomic DNA from water samples, which have been cultured but did not give isolates for *H. pylori*, were prepared by centrifuging 1 mL of the liquid portion of slant MCUA tube at 14000 ×g for 2 min and washed with 1 mL of PBS to be completed by the same steps for *H. pylori* isolates. Concentration and purity were measured spectrophotometrically at OD_260_ and OD_280_ respectively, to exclude any possible contamination, and a gel of 0.8% agarose was used for electrophoresis.

### 2.7. PCR Amplification of 16SrRNA for H. pylori

Amplification was carried out in a 25 *μ*L of reaction mixture containing 12.5 *μ*L master mix, 0.5 *μ*L forward primer, 0.5 *μ*L reverse primer, 5 *μ*L DNA samples, 6.5 *μ*L distilled water and 25 *μ*L mineral oil. PCR conditions for 16SrRNA include: denaturation step at 95°C for 5 min, followed by 39 cycles at 94°C for 1 min, annealing at 55°C for 1 min and extension at 72°C for 2 min, and an additional extension step at 72°C for 7 min. PCR products were electrophoresed in 2% agarose. 

### 2.8. ureA Gene for H. pylori and PCR Amplification

All isolates which were confirmed by 16SrRNA have been tested for the presence of the ureA gene of *H. pylori*. The primer for 411 bp product of the ureA sequence represented by the forward primer sequence: 5 GCC AAT GGT AAA GCC TTA GTT3 and the reverse one: 5 CTC CTT AAT TGT TTT TAC 3 [17]. Amplification was carried out in a 25 *μ*L of reaction mixture containing 12.5 *μ*L master mix, 0.5 *μ*L forward primer, 0.5 *μ*L reverse primer, 5 *μ*L DNA samples, 6.5 *μ*L distilled water and 25 *μ*L mineral oil. PCR conditions for ureA gene include: denaturation step at 95°C for 5 min, followed by 35 cycles at 94°C for 1 min, annealing at 45°C for. 1 min and, extension at 72°C for 1 min and an additional extension step at 72°C for 7 min. PCR products were electrophoresed in 2% agarose. 

## 3. Results

### 3.1. Culture Results

 Out of 471 water samples, 14 (2.76%) isolates of *H. pylori* were isolated from samples taken from 14 districts by culture method and identified by biochemical tests. They consist of 11 (4.13%) *H. pylori* that have been isolated and diagnosed from 266 samples of tap water and 3 ones (1.46%) from 205 RO samples.

**Figure 1:**
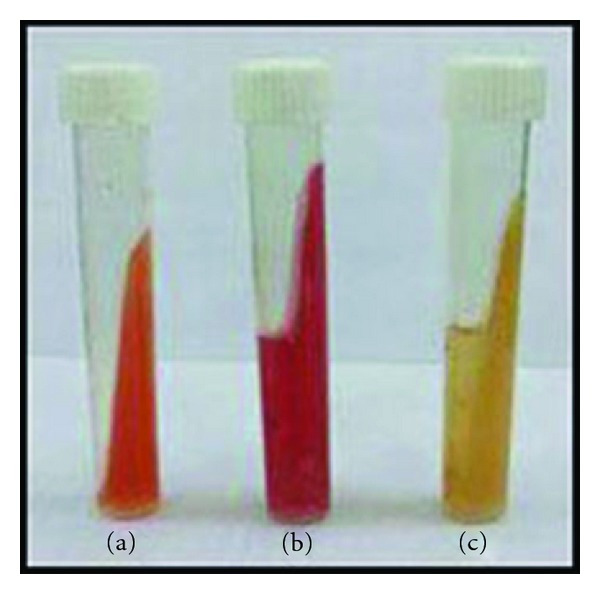
Change in color of slant MCUA tube, (a) slant MCUA tube only, (b) positive slant MCUA tube, culture, (c) negative slant MCUA tube, culture.

The modified Columbia urea agar using MDCS method preliminarily revealed the presence of* H. pylori* in water samples, correlated with the change in the color of the slant MCUA tube from orange to pink that occurred at the same time thus giving an additional evidence for the presence of *H. pylori* in the samples (Figure 1).

The isolation rate upon subculturing on HP medium was 14/471 (2.76%) isolates of *H. pylori*, while on MCUA medium was 6/471 (1.2%) isolates included in the 14 isolates of *H. pylori*.

On MCUA medium, the colonies of the isolated *H. pylori* were small to middle in size, rounded, and creamy in color, while, on HP medium, the isolated *H. pylori* were small in size, rounded, and transparent. Both the MCUA and HP media showed change in color from yellow/orange to red.

All *H. pylori* isolates were Gram-negative spiral to coccobacilli and shared the characteristic catalase, urease, and oxidase production, but differ slightly with respect to other tests (Table 1). Collectively, 3 isolates are being positive in nitrate reduction, 2 in being able to grow at 42°C, and 9 negatives in both traits. 

### 3.2. Antibiotics Susceptibility

**Figure 2:**
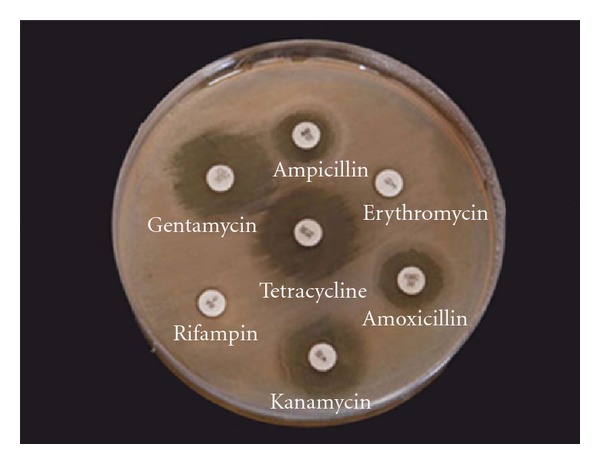
Antibiotic effects on *H. pylori* isolated from drinking water.

For *H. pylori* isolates from drinking water, tetracycline was found to be the most effective antibiotic, 71% of the tested isolates were sensitive to tetracycline followed by kanamycin 57% and gentamycin 36%, ampicillin 14%. Rifampicin and amoxicillin were shown to be the least effective ones (7%) against *H. pylori* isolated from drinking water,while erythromycin was a non effective antibiotic, as shown in (Figure 2) and in reference to interpretive chart of zone sizes.

### 3.3. PCR Results

Only 6 out of 11 (54.5%) *H. pylori* morphologically and biochemically identified isolates from tap water were found to harbor 16SrRNA gene and of the 3 R.O isolates only one (33.3%) isolate gave positive results for 16SrRNA gene by PCR. Thus leaving out 50% of the conventionally identified isolates as false positive. From the100 samples negative for* H. pylori *by culturing, only 4 (4%), gave positive results for 16SrRNA. 

**Figure 3:**
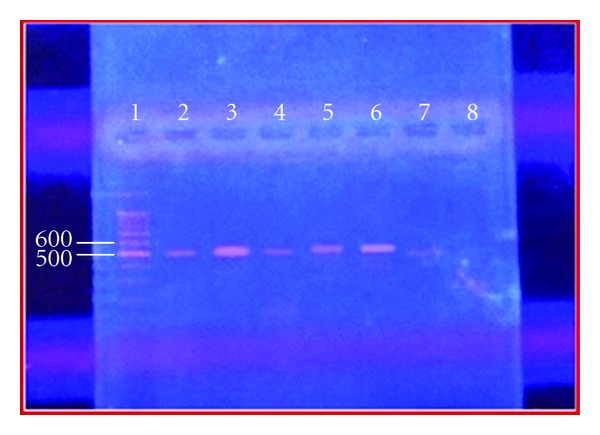
PCR products for 16SrRNA-based primers gave band on agarose gel corresponding to a 500 base pair product when compared to the molecular ladder. Lane 1, molecular ladder (1500–100) bp, lane (2–6) bands of PCR products for *H. pylori* with 16SrRNA.

PCR products for 16SrRNA based primers gave bands on agarose gel corresponding to a 500 base pair product when compared to the molecular ladder, thus identifying the isolates as *H. pylori* as shown in (Figure 3). 

### 3.4. ureA Gene for H. pylori and PCR Amplification

**Figure 4:**
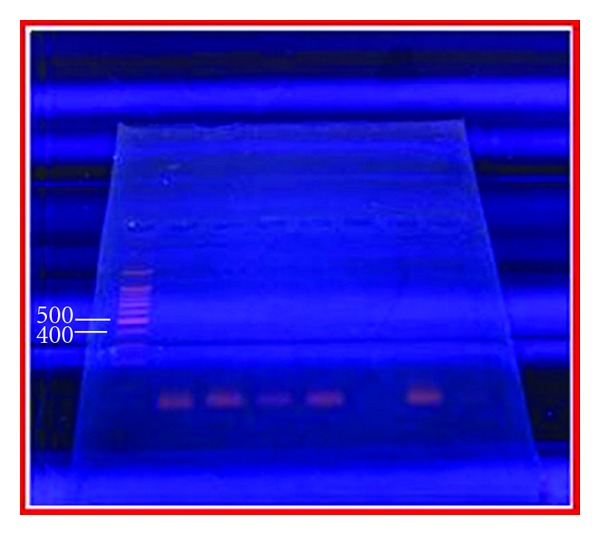
PCR product for *H. pylori* with ureA gene based primers. Lane 1, molecular ladder (1500–100) bp, no band of PCR product for *H. pylori* with ureA gene have been obtained.

All isolates of *H. pylori* which have been confirmed by 16SrRNA, did not give specific results to ureA (Figure 4), only products of 100 bp have been obtained and also a much larger bands. 

## 4. Discussion

Natural habitat of *H. pylori* is in the human stomach, other sources of *H. pylori* and its mode of transmission are unknown [18]. In this study, *H. pylori* has been isolated and diagnosed from drinking water by culture method and a combination of biochemical and PCR test. The first indication for the presence of *H. pylori* in water came from AL-Sulami et al. [8] in which 10 isolates were identified as *H. pylori* by biochemical tests. That finding has been confirmed by current study using the same method and a combination of conventional and PCR tests in identifying recovered *H. pylori*.

A low recovery of a pathogen is not surprising considering various factors affecting its survival in water. Upon primary, isolation there were 78 urease-positive isolates obtained from 266 tap water samples and 43 urease-positive ones from RO water samples. The numbers were reduced to 11 and 3 isolates, respectively, after subjecting them to conventional tests leaving 67 and 40 false-positive ones. Urease-negative isolates were not considered. Other bacteria were mainly pseudomonads.

So far there is no published paper proving the viability of coccoid form or the possibility that coccoid form transforms to spiral bacillary form. Our results indicate that some *H. pylori* are still viable and appear as spiral bacillary after Gram staining smears from colonies on MCUA; others are not and only can be detected by PCR.

Based on the assumption that all *H. pylori* in drinking water are coccoid [6], the results implicitly indicate the possibility of the transformation of some coccoid form to spiral bacillary form.

 It is difficult to compare our data with those published, because each author has used a distinct method to detect the bacterium, and all attempts to culture the organism directly from water samples [18, 19] have been unsuccessful. This may be due to the fact that overgrowth by other microorganisms on the rich media led to the difficulty of isolation of *H. pylori* from water, and another reason for the lack of recovery of *H. pylori* from the environment is the fastidious nature of *H. pylori* which has a polymorphisms phenomenon. Under these circumstances, the organism would not be recovered by traditional culture techniques; hence in our study we developed a different protocol for culturing *H. pylori* from water. The importance of this method is to provide a possibility of successful culture method for *H. pylori*.

In general, high-resistance profile to the tested antibiotics is apparent on these isolates as indicated by *H. pylori* resistance for tetracycline in 29% of the isolates, also in case of kanamycin *H. pylori* resistance of 43% which is less than Al-Sulami et al. [8] result of 60%. Amoxicillin which represents active antibiotics in treatment of this bacterium was ineffective with a resistance in 93% of the isolates.

### 4.1. 16SrRNA for H. pylori Detection by PCR

In this study, this is the first report on using 16SrRNA amplification and confirmation of *H. pylori* isolates from environmental samples in Iraq. The 16SrRNA was chosen for detection of *H. pylori* because it exhibits a high degree of functional and evolutionary homology within all bacteria [9]. Only 7 isolates, out of 14 morphologically and biochemically identified *H. pylori*, were confirmed by 16SrRNA as they gave positive results for 16SrRNA. The prevalence of false positive isolates by conventional tests indicates a nonspecific approach. Meanwhile, in 100 drinking water samples in which no *H. pylori* was detected by culture method, 4 samples produced positive results by 16SrRNA. This means that cells of *H. pylori* that are not detected by culture method can be done by PCR, and hence, the MDCS provides the opportunity for simultaneous detection of both culturable and nonculturable forms.

Results of PCR products of 16SrRNA gene amplification revealed the presence of 500 base pair sequence of the gene coded the 16SrRNA molecule, and this result agrees with that of [15]. The size of PCR product was determined by comparing it with a DNA ladder, which contains DNA fragments of known size (1500–100) base pairs. Our results may shed additional light on the evidence supporting water-borne transmission which emanates from the fact that there is a direct recovery of *H. pylori* from tap water and R.O water concomitantly confirmed by PCR.

### 4.2. ureA Gene for H. pylori Detection by PCR

The ureA genotype was expected to be present in all *Helicobacter* positive strains. However, our study was unable to detect the ureA gene in the isolates of *H. pylori* already confirmed by 16SrRNA. This result agrees with Tiveljung et al. [20] who used ureA gene and were unable to detect it in *H. pylori* strain regarded as normal control.

## Figures and Tables

**Table 1:** Results of biochemical tests characterizing *H. pylori* isolates from 14 districts.

District no	Catalase	Oxidase	Urease	Nitrate reduction	H_2_S	Growth with 3.5% NaCl	Growth on 1% glycin	Growth at 42°C	Growth at 25°C
1	+	+	+	−	−	−	−	−	−
2	+	+	+	−	−	−	−	+	−
3	+	+	+	+	−	−	−	−	−
4	+	+	+	−	−	−	−	−	−
5	+	+	+	−	−	−	−	+	−
6	+	+	+	+	−	−	−	−	−
7	+	+	+	−	−	−	−	−	−
8	+	+	+	−	−	−	−	−	−
9	+	+	+	−	−	−	−	−	−
10	+	+	+	−	−	−	−	−	−
11	+	+	+	+	−	−	−	−	−
12	+	+	+	−	−	−	−	−	−
13	+	+	+	−	−	−	−	−	−
14	+	+	+	−	−	−	−	−	−
